# Clinical characteristics of chronic central serous chorioretinopathy patients with insufficient response to reduced-settings photodynamic therapy

**DOI:** 10.1007/s00417-018-4003-z

**Published:** 2018-05-07

**Authors:** Thomas J. van Rijssen, Elon H. C. van Dijk, Greet Dijkman, Camiel J. F. Boon

**Affiliations:** 1 Department of Ophthalmology, Leiden University Medical Center, Albinusdreef 2, 2333 ZA Leiden, The Netherlands; 2 Department of Ophthalmology, Academic Medical Center, University of Amsterdam, 22660, 1100 DD Amsterdam, The Netherlands

**Keywords:** Chronic central serous chorioretinopathy, Optical coherence tomography, Photodynamic therapy, Resolution, Subretinal fluid, Treatment response

## Abstract

**Purpose:**

To identify characteristics of Caucasian chronic central serous chorioretinopathy (cCSC) patients without a complete resolution of subretinal fluid (SRF) after reduced-settings photodynamic therapy (PDT), or with a recurrence of SRF after PDT.

**Methods:**

Chronic CSC patients treated with reduced-settings PDT were divided into a successful PDT group and unsuccessful PDT group. Patients in the successful PDT group did not have any subretinal fluid (SRF) during follow-up after PDT, whereas the unsuccessful PDT group was categorized based on either persistence or recurrence of SRF after PDT treatment. Data on age, sex, best-corrected visual acuity (BCVA), PDT spot size, characteristics on fluorescein angiography (FA), indocyanine green angiography (ICGA), and optical coherence tomography (OCT) were obtained.

**Results:**

Twenty-six patients in the successful PDT group (20 males, 6 females) had a mean age of 51 years (range, 25–78). In the unsuccessful PDT group, 20 males with a mean age of 60 years (range, 34–78) were included. At last visit before PDT, age, percentage of males, and percentage of patients with diffuse leakage > 1 optic disc diameter on FA were higher in the unsuccessful PDT group (*p* = 0.010, *p* = 0.029, and *p* = 0.008, respectively). At last visit before PDT, BCVA and the percentage of patients with intense hyperfluorescence on ICGA were lower in the unsuccessful group (*p* = 0.017 and *p* = 0.004, respectively). Patients with intense hyperfluorescence on ICGA were more likely (95% CI 1.3–333 times) to have a successful outcome (*p* = 0.045). A decrease in SFCT at final visit was observed in both groups (− 111 μm and *p* = 0.013, and − 141 μm and *p* = 0.007, respectively). BCVA only improved at final visit in the successful PDT group (5 Early Treatment of Diabetic Retinopathy Study letters, *p* < 0.001).

**Conclusions:**

Chronic CSC patients with recurrent or persistent SRF after PDT are characterized by a higher percentage of males, more patients with diffuse leakage on FA, more patients without intense hyperfluorescence on ICGA, higher age, and lower pre-PDT and long-term BCVA than in the successful PDT group. A reduction in SFCT after PDT does not necessarily lead to complete resolution of SRF, while a resolution of SRF appears to be required to lead to a significant BCVA improvement in cCSC.

## Introduction

Central serous chorioretinopathy (CSC) is a chorioretinal disease characterized by an accumulation of subretinal fluid (SRF), which often affects the macula, with subsequent central vision loss and decreased quality of life [1, 2]. Abnormalities in the retinal pigment epithelium (RPE) and choroid are primarily involved in CSC pathogenesis, but the exact cause of the disease is unclear [2–4]. Choriocapillaris hypoperfusion with hyperperfusion of the surrounding arterioles and venules can be seen in chronic CSC (cCSC) patients [5, 6]. Middle- and late-phase indocyanine green angiography (ICGA) can reveal abnormalities of the choroid, visible as hyperfluorescent areas typical of CSC [6, 7]. CSC mainly affects middle-aged male individuals, while other risk factors include the use of corticosteroids, possibly type A personality, and pregnancy [2, 8]. CSC is often subcategorized into 2 broad categories: acute CSC and cCSC. In acute CSC, SRF usually resolves spontaneously within a few months [2]. This is in contrast with the persistent SRF and often more extensive retinal and choroidal abnormalities in cCSC, which can lead to permanent photoreceptor damage and visual dysfunction [4]. Photodynamic therapy (PDT) has been shown to be an effective treatment option for cCSC, also when using reduced settings in comparison with the original settings described for neovascular age-related macular degeneration [9–12]. Several types of PDT with reduced settings have been developed: half-dose PDT, half-fluency PDT, and half-time PDT, for which no significant differences in treatment outcome have been found thus far [9, 13, 14]. The mechanism of action in PDT in CSC is thought to be short-term choriocapillaris hypoperfusion and subsequent choroidal vascular remodeling, with cessation of leakage to the subretinal area, despite the damaged outer blood-retinal barrier of the RPE [15, 16]. Although SRF resolves completely after PDT in a high percentage of cCSC patients (62–100%), the desired treatment effect of a complete SRF resolution will not be attained in every case [17–19], and some patients develop a recurrence of SRF after initial SRF resolution after PDT treatment [20].

Relatively, little is known about clinical parameters that are associated with the outcome of PDT in cCSC. In a study of Asian cCSC patients, a lower baseline visual acuity and higher age before PDT have recently been described to be associated with persistence and recurrence of SRF after half-dose PDT [21]. Also, the degree of hyperfluorescence on ICGA and the presence of posterior cystoid retinal degeneration may influence PDT outcome [18, 22]. The aim of our study is to identify clinical characteristics associated with either incomplete or no response to PDT in Caucasian cCSC patients, and to describe treatment outcome in patients with an incomplete or no response to PDT.

## Methods

Patients were included retrospectively, based on the presence of SRF on optical coherence tomography (OCT), at least either 1 area of diffuse leakage, 1 “hot spot” of leakage, or irregular RPE window defects on fluorescein angiography (FA), with corresponding hyperfluorescent abnormalities on ICGA.

A disease period defined as vision loss due to persistently active SRF leakage in the macula of more than 3 months had to have been present. All included patients were Caucasian and received PDT in LUMC between November 2011 and February 2016. Included patients were divided into 2 groups, based on outcome of PDT treatment. A successful PDT was defined as complete resolution of SRF on OCT after PDT, and no recurrence of SRF during follow-up. An unsuccessful PDT was defined as either no complete resolution of SRF within 2 months after PDT or a recurrence of SRF on OCT after PDT treatment during follow-up. For both groups, follow-up had to be available for at least until 1 year after first PDT in LUMC. Patients with either myopia of more than 6 dioptres, suspicion of either choroidal neovascularization, or evidence of other diseases than CSC as a cause of SRF or patients without the aforementioned multimodal imaging available at baseline were excluded.

The PDT procedure started with an intravenous infusion of either 3 mg/m^2^ (half dose) or 6 mg/m^2^ (full dose) verteporfin (Visudyne®; Novartis, Basel, Switzerland), in 10 min. An anesthetic eye drop was administered (oxybuprocaine 0.4% or equivalent) to the affected eye at 15 min after the start of infusion, and a contact lens was positioned on the anesthetized eye. Subsequently, laser therapy with standard fluency of either 25 J/cm^2^ (half fluency) or 50 J/cm^2^ (full fluency), and a wavelength of 689 nm was applied to the affected area for either 42 (half time) or 83 s (full time). Patients who were treated with half-dose PDT received laser therapy with full fluency and full time. Patients treated with half-fluency PDT received a full dose of verteporfin and a full-time laser treatment. In the half-time PDT group, patients were treated with full-dose verteporfin and full fluency. The area to be treated was based on hyperfluorescent changes on ICGA. If a single laser spot did not cover the complete hyperfluorescent area or multifocal hot spots of leakage were present, additional spots could be applied.

Included patients visited the outpatient clinic of the Department of Ophthalmology of Leiden University Medical Center. They underwent Snellen best-corrected visual acuity (BCVA) measurement, which was later converted to Early Treatment of Diabetic Retinopathy Study (ETDRS) letters according to a previously published protocol, for statistical analysis [23]. Patients received an extensive ophthalmological examination including slit lamp examination, fundoscopy, and either time-domain OCT (Cirrus HD-OCT; Carl Zeiss Meditec; or OCT-HS100; Canon Inc., Tokyo, Japan) or spectral-domain OCT (Spectralis HRA+OCT; Heidelberg Engineering, Heidelberg, Germany) at each visit. FA was performed at least once, and ICGA was obtained when available (either with Topcon Corp., Spectralis HRA+OCT, or Carl Zeiss Meditec). Patient data from last visit before PDT, first visit after PDT, first visit after second PDT (if applicable), and last visit were studied. At last visit before PDT, data on age, sex, refractive error, medical history, and medication use were collected. BCVA, central foveal thickness (CFT), subfoveal choroidal thickness (SFCT), and the presence of SRF and posterior cystoid retinal degeneration were evaluated during each visit.

In order to obtain CFT, the distance between the inner limiting membrane and the inner part of the ellipsoid zone was measured manually in the foveal pit with the build-in caliper tool of the OCT machine software. The foveal pit was recognized by maximal foveal dip, hyperreflectivity of the inner limiting membrane, and shape of the outer nuclear layer. SFCT was measured manually on enhanced-depth imaging OCT between the choroidal and scleral layers, when the image was of sufficient quality. The type of pigment epithelial detachment (PED) that was present in patients was categorized conforming to a previous study protocol [24]. Patients were categorized based on the presence of a regular PED with hyporeflectivity inside the PED, RPE bumps, or irregular PED including hyperreflectivity inside the PED on OCT. Diffuse leakage larger than 1 optic disc diameter was defined as a hyperfluorescent area of leakage larger than 1 optic disc diameter without a clear leakage focus within the temporal vascular arcades on 3 min FA. Hyperfluorescent abnormalities on FA were considered to be RPE alterations without active leakage when there was no increase in size between early-phase FA and late-phase FA. The degree of hyperfluorescence on ICGA was assessed, based on the criteria reported in previous studies [18, 22]. This degree of hyperfluorescence was categorized in intense hyperfluorescence, mild hyperfluorescence, or absence of hyperfluorescence on 10-min ICGA. The area that was assessed on ICGA was overlapping with the area of the hot spot on FA and SRF on OCT.

For statistical analysis, paired *t* tests were performed using IBM SPSS statistics software for Windows, version 23 (IBM Corp., Armonk, NY, USA), within the successful and unsuccessful PDT groups. Independent *t* tests were performed to compare the successful and unsuccessful PDT groups at baseline. A binary logistic regression analysis was performed to ascertain the effects of age, sex, BCVA at last visit before PDT, diffuse leakage larger than 1 optic disc diameter on FA, degree of hyperfluorescence on ICGA, posterior cystoid degeneration, and spot size of first PDT in the LUMC on the likelihood that patients have an unsuccessful outcome to PDT. Subgroup analyses were performed within the unsuccessful PDT group. In these subgroup analyses, patients with no resolution of SRF and with recurrent SRF after a previous complete resolution of SRF were included.

## Results

### Patient characteristics

Out of the 451 patients diagnosed with CSC, 46 patients could be included in this study. These 40 male and 6 female patients had a mean age of 55.1 ± 11.4 years. Out of the included patients, 41 received half-dose PDT, 4 patients received reduced-fluency PDT, and 1 patient received half-time PDT. The mean spot size of the PDT was 5189 ± 2002 μm. The mean duration from date of CSC diagnosis until first PDT in LUMC was 3.4 ± 5.2 years. The mean duration from PDT to first visit after PDT was 7.3 ± 2.3 weeks. The time from first PDT until last evaluation visit was 25.7 ± 14.6 months.

### Characteristics of successful and unsuccessful PDT groups

**Fig. 1:**
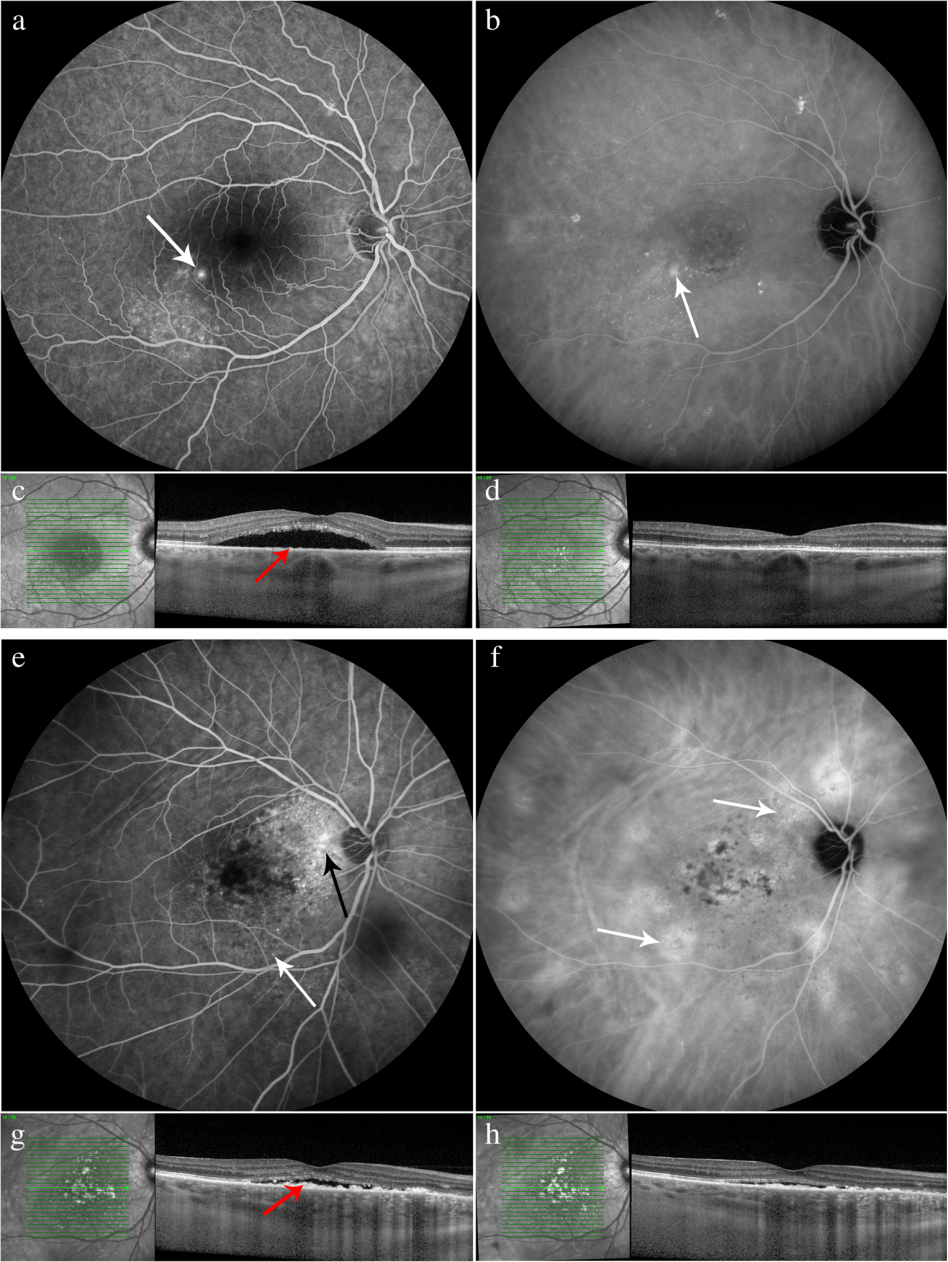
Characteristics on multimodal imaging of 2 chronic central serous chorioretinopathy (cCSC) patients, both treated with reduced-settings photodynamic therapy (PDT). Fluorescein angiography (FA; **a**), indocyanine green angiography (ICGA; **b**), and optical coherence tomography (OCT; **c**) at last visit before PDT and OCT at first visit after PDT (**d**) of a 52-year-old male cCSC patient with a successful outcome to PDT. At first visit after PDT, subretinal fluid on OCT has resolved (**d**). A “hot spot” of leakage is present on FA (white arrow; **a**) at last visit before PDT. Inferiorly and temporally of the fovea, retinal pigment epithelium (RPE) alterations are detected, whereas ICGA imaging shows hyperfluorescent abnormalities within the vascular arcades (**b**, white arrow). On OCT scan (**c**), minor RPE bumps are visible (red arrow). FA (**e**), ICGA (**f**), and OCT (**g**) at last visit before PDT and OCT at first visit after PDT (**h**) of a 62-year-old male cCSC patient with an unsuccessful response to PDT. At first visit after PDT, subretinal fluid is still present on OCT (**h**). Diffuse leakage is visible on FA (**e**, black arrow), and RPE alterations are present (**e**, white arrow). On ICGA (**f**, white arrows), hyperfluorescent and hypofluorescent abnormalities are visible. ICGA images clearly show eccentric multifocal ill-defined areas of hyperfluorescent abnormalities typical of cCSC, but the central area treated by PDT shows relatively sparse hyperfluorescence. On OCT scan (**g**), irregular hyperreflective thickening of the RPE layer is present (red arrow)

PDT treatment was successful in 26 patients. An unsuccessful PDT outcome occurred in 20 patients: an incomplete resolution of SRF occurred in 16 patients and recurrence of SRF in 4 patients. Multimodal imaging of both a patient who received successful PDT and a patient in whom PDT proved to be unsuccessful is shown in Fig. 1. Out of the patients with an unsuccessful PDT, 10 received a second PDT treatment with a mean spot size of 5070 ± 2074 μm. The only patient that received half-time PDT and 1 out of the 4 patients that received reduced-fluency PDT, had an unsuccessful PDT outcome. The mean duration between the first PDT treatment and second PDT treatment was 10.6 months (range, 1.6–32.8). In the unsuccessful PDT group, 3 patients had previously received PDT treatment with no successful initial result at another clinic, whereas none of the patients in the successful group were previously treated with PDT. In the subgroup of 4 patients who had a recurrence of SRF after initial complete resolution after PDT, 3 patients showed hyperfluorescent abnormalities on ICGA before PDT treatment. Extrafoveal recurrences were seen in these 3 patients with hyperfluorescent abnormalities on ICGA. In 2 patients, these areas with extrafoveal recurrences showed overlap with areas that were affected by SRF prior to PDT, and 1 patient had a recurrence of SRF at an area that was not previously affected by SRF. The mean time from first PDT until recurrence of SRF was 21.3 ± 7.4 months. The characteristics of both the successful and unsuccessful PDT groups are depicted in Table 1. There were no significant differences in SFCT, CFT, presence of posterior cystoid retinal degeneration, RPE alterations larger than 5 optic disc diameters, PED or irregular RPE, and spot size between the groups. Significant differences between the 2 groups were noted, including a significant difference in age (59.9 ± 12.1 years in the unsuccessful group versus 51.4 ± 9.5 years in the successful group, *p* = 0.010), percentage of males (100% in the unsuccessful group and 77% in the successful group, *p* = 0.029), BCVA before PDT (65.6 ± 22.0 ETDRS letters in the unsuccessful group versus 78.3 ± 12.2 ETDRS letters in the successful group, *p* = 0.017), percentage of patients with intense hyperfluorescence on ICGA (45% in the unsuccessful group versus 91% in the successful group, *p* = 0.004), and more patients with diffuse leakage larger than 1 optic disc diameter (35% in the unsuccessful group versus 4% in the successful group, *p* = 0.008). Secondary outcomes are summarized in Table 2.

**Table 1:** Characteristics of chronic central serous chorioretinopathy patients in the unsuccessful and successful PDT groups at baseline

	Unsuccessful PDT (*n* = 20)	Successful PDT (*n* = 26)	
	Mean ± SD	Mean ± SD	*p* value
Age at PDT, in years	59.9 ± 12.1	51.4 ± 9.5	0.010*
Duration from CSC diagnosis to PDT, in years	4.6 ± 6.4	2.4 ± 3.9	0.157
Duration from PDT to last follow-up visit, in months	28.5 ± 14.9	23.5 ± 14.3	0.258
BCVA in ETDRS	65.6 ± 22.0	78.3 ± 12.2	0.017*
SFCT, in μm (missing)	476.8 ± 145.5 (8)	403.6 ± 174.5 (10)	0.250
CFT, in μm (missing)	98.7 ± 28.0 (7)	106.8 ± 19.8 (9)	0.362
Spot size PDT, in μm	5280 ± 1476	5116 ± 2369	0.788
	Number; percentage	Number; percentage	*p* value
Male gender	20; 100%	20; 77%	0.029*
Presence of posterior cystoid retinal degeneration	7; 35%	6; 23%	0.511
RPE alterations larger than 5 optic disc diameters on FA	9; 45%	6; 23%	0.204
Diffuse leakage larger than 1 optic disc diameter on FA	7; 35%	1; 4%	0.008*
Intense hyperfluorescent abnormalities on ICGA (missing)	9; 45% (0)	20; 91% (4)	0.004*
Pigment epithelial detachment (PED)			0.488
- Regular PED	4; 20%	3; 12%	
- RPE bump	6; 30%	11; 42%	
- Irregular PED	10; 50%	12; 46%	

*BCVA* best-corrected visual acuity, *CFT* central foveal thickness, *ETDRS* Early Treatment of Diabetic Retinopathy Study, *FA* fluorescein angiography, *ICGA* indocyanine green angiography, *OCT* optical coherence tomography, *PDT* photodynamic therapy, *SD* standard deviation, *SFCT* subfoveal choroidal thickness, *RPE* retinal pigment epithelium

*p values < 0.05 were considered statistically significant

**Table 2:** Clinical parameters in chronic central serous chorioretinopathy patients during follow-up in the unsuccessful and successful PDT-treated groups

	Unsuccessful PDT				
	Last visit before PDT	First visit after PDT		Final visit	
	Mean ± SD (number)	Mean ± SD (number)	*p* value	Mean ± SD (number)	*p* value
BCVA in ETDRS letters	66 ± 22 (20)	71 ± 20 (20)	0.012*	69 ± 22 (20)	0.062
SFCT in μm	477 ± 145 (12)	368 ± 109 (13)	0.029*	336 ± 81 (15)	0.007*
CFT in μm	99 ± 28 (13)	105 ± 31 (14)	0.234	110 ± 36 (18)	0.143
	Successful PDT				
	Last visit before PDT	First visit after PDT		Final visit	
	Mean ± SD (number)	Mean ± SD (number)	*p* value	Mean ± SD (number)	*p* value
BCVA in ETDRS letters	78 ± 12 (26)	81 ± 12 (26)	0.022*	83 ± 12 (27)	< 0.001*
SFCT in μm	421 ± 190 (13)	320 ± 93 (12)	0.044*	310 ± 122 (13)	0.013*
CFT in μm	107 ± 20 (17)	113 ± 19 (16)	0.044*	115 ± 25 (17)	0.151

*BCVA* best-corrected visual acuity, *CFT* central foveal thickness, *ETDRS* Early Treatment of Diabetic Retinopathy Study, *PDT* photodynamic therapy, *SD* standard deviation, *SFCT* subfoveal choroidal thickness; *p* values compared to last visit before PDT

*p values < 0.05 were considered statistically significant

In the successful PDT group, we observed an average increase of 2.4 ± 5.1 ETDRS letters (*p* = 0.022), mean CFT increase of 7.1 ± 12.8 μm (*p* = 0.044), and a mean SFCT decrease of 102.1 ± 155.7 μm (*p* = 0.044) at first visit after PDT, compared to last visit before PDT. At final visit, BCVA was significantly higher (mean = + 4.8 ± 5.7 ETDRS letters, *p* < 0.001), CFT was higher (mean = + 7.9 ± 21.5 μm, *p* = 0.151), and SFCT was lower (mean = − 111.3 ± 137.6 μm, *p* = 0.013) compared to last visit before PDT. In the unsuccessful PDT group, BCVA was higher (mean = + 5.9 ± 9.4 ETDRS letters, *p* = 0.012), CFT was on average 5.8 ± 16.8 μm (*p* = 0.234) higher, and SFCT was significantly lower (mean = − 108.8 ± 150.2 μm, *p* = 0.029) at first visit after PDT, compared to last visit before PDT. At final visit, ETDRS was higher (mean = + 3.8 ± 8.6 ETDRS letters, *p* = 0.062), CFT was higher (mean = + 11.8 ± 27.0 μm, *p* = 0.143), and SFCT was significantly lower (mean = − 141.3 ± 147.5 μm, *p* = 0.007) than at last visit before PDT.

### Binary logistic regression model

The binary logistic regression model that was computed was statistically significant (chi-square 26.966 (8), *p* < 0.001). The model explained 63% of the variance (Nagelkerke *R*^2^) and correctly classified 81% of the patients. Patients with intense hyperfluorescence on ICGA were 22 times more likely to have a successful PDT outcome (95% CI 1.3–333, *p* = 0.045). Age (*p* = 0.965), sex (*p* = 0.999), BCVA at last visit before PDT (*p* = 0.670), diffuse leakage on FA (*p* = 0.082), presence of posterior cystoid retinal degeneration (*p* = 0.391), and spot size (*p* = 0.428) were not significantly associated with PDT outcome.

## Discussion

The aim of our study was to compare the characteristics of Caucasian patients with a successful PDT response, defined as complete resolution of SRF on OCT after PDT, to characteristics of patients with an unsuccessful PDT response, defined as the presence of SRF within 2 months after PDT, or a recurrence of SRF after PDT. Based on the results of our study, we conclude that PDT treatment in Caucasian cCSC patients without hyperfluorescent areas on ICGA is less likely to respond with a resolution of SRF, and that the rate of recurrence in these patients may be high. Long-term BCVA prognosis may be worse in patients with an unsuccessful outcome to PDT compared to patients with a successful outcome to PDT.

BCVA, degree of hyperfluorescence on ICGA, posterior retinal cystoid degeneration, and age at the time of PDT have previously been associated with the outcome of PDT in cCSC patients [18, 21, 22, 25]. In the regression model used in our study, we were only able to verify the association between the degree of hyperfluorescence on ICGA and PDT outcome. This may be due to the possibility that the previously described parameters are associated with hyperfluorescent abnormalities on ICGA, or the limited number of patients that were included. Nevertheless, we did find significant differences in age, sex, BCVA at last visit before PDT, percentage of patients with diffuse leakage, and degree of hyperfluorescence on ICGA between the successful and unsuccessful PDT groups.

Choroidal thickening, leakage, and congestion, as well as RPE dysfunction, have been described to be involved in cCSC [3, 26, 27]. PDT affects the choroid by presumably inducing vascular remodeling and a reduction in leakage of fluid from the choriocapillaris, thus reducing fluid leakage through the dysfunctional RPE outer blood-retinal barrier. In patients with an unsuccessful PDT outcome, SRF accumulation persisted despite a significant reduction in SFCT that was comparable to that in the successful PDT group. A significant SFCT is therefore not necessarily sufficient to lead to SRF resolution. Such persistent fluid leakage despite an effect on SFCT may be explained by more severe RPE abnormalities and therefore more pronounced outer blood-retina barrier dysfunction that outweighs the PDT effects on the choroid [27]. In the patients in this unsuccessful PDT group, one could hypothesize that PDT is not successful because there is less involvement of active leakage and congestion of the choroid, as evidenced by for instance the presence of hypofluorescence or only mild hyperfluorescence on ICGA or mildly increased SFCT. However, in this study, we did not find a significant difference in SFCT between the successful and unsuccessful PDT groups. In our study, we found a positive association between intense hyperfluorescence on ICGA and successful PDT outcome. However, we did not find a significant difference in SFCT and PED category between the successful and unsuccessful PDT groups at last visit before PDT. In a previous Asian cCSC patient cohort, PDT has also been described to be less effective and with a higher recurrence rate in patients without intense hyperfluorescence on ICGA [22]. Apparently, a more hyperfluorescent, leaky choriocapillaris is more sensitive to the remodeling treatment effects of PDT and can be predictive of a favorable treatment response [28].

Inoue et al. have suggested that an imbalance between choroidal hydrostatic pressure and RPE integrity can explain recurrences of SRF [22]. In the unsuccessful PDT group, we found a higher percentage of patients with diffuse leakage larger than 1 optic disc diameter at last visit before PDT. In patients with diffuse leakage on FA, the RPE outer blood-retina barrier (and possibly also the choroid) may be relatively more damaged, which could influence the treatment effect of PDT. In the elderly patient with prolonged CSC, the choriocapillaris may eventually become less susceptible to PDT-mediated remodeling than in younger patients, despite a persistent relatively thickened underlying Haller and Sattler layer containing the larger, unfenestrated choroidal vessels [29, 30].

Previous studies have shown that a prolonged presence of SRF may lead to permanent damage to photoreceptor cells, resulting in a decreased BCVA that is at least partially irreversible [31–33]. Therefore, PDT treatment in an early stage of cCSC in order to reduce the negative effects of the presence of SRF may lead to improved functional and anatomical treatment outcome [21]. Since duration of the presence of SRF may be associated with BCVA, we may suspect a longer duration of SRF in the unsuccessful PDT group. We did not find a significant longer duration of SRF in the unsuccessful PDT group compared to the successful PDT group in our study, which may be due to the small number of included patients. In the successful PDT-treated group, we found a significant increase in BCVA at final visit. In the unsuccessful PDT group, there was a significant increase in BCVA only at first visit after PDT. The recurrence of SRF and an accompanying decrease in BCVA in some patients may partially explain this observation.

This study has limitations. The duration of follow-up of patients included in this study was limited due to the retrospective nature of this study. Moreover, a selection bias may have occurred, since some of our patients may have had a recurrence of SRF after available follow-up, which had to be at least 1 year. Since there was no significant difference in duration of follow-up between both groups, the possible effect of this bias has been minimalized. One could argue that patients with a recurrence of SRF, who are a subgroup of the unsuccessful PDT group in this study, should be considered to have had a successful outcome because of the long absence of SRF, for an average of 19 months. However, recurrent SRF is also presumed to lead to progressive visual deterioration [1]. Since we did not perform OCT angiography in this study, we cannot completely exclude the presence of subtle type I neovascularization or branching vascular network in the included patients. However, there was no sign of neovascularization on OCT, FA, and ICGA in the study population.

To the best of our knowledge, this is the first evaluation of the characteristics of Caucasian cCSC patients that evaluates reduced-settings PDT treatment outcome. According to our results at the visit before PDT and final visit, successful PDT leads to an increase in BCVA and decrease in SFCT, whereas unsuccessful PDT only leads to a decrease in SFCT. Absence of intense hyperfluorescence on ICGA is associated with a less favorable response to reduced-settings PDT.
